# Learning with self-generated drawings and the impact of learners’ emotional states

**DOI:** 10.3389/fpsyg.2023.1286022

**Published:** 2023-11-16

**Authors:** Sabrina D. Navratil, Tim Kühl

**Affiliations:** ^1^Psychology of Education, University of Mannheim, Mannheim, Germany; ^2^Institute for Psychology of Learning and Instruction, Kiel University, Kiel, Germany

**Keywords:** multimedia learning, generative learning strategies, drawing, emotional states, motivation, time on task

## Abstract

**Introduction:**

The present study examined the influence of emotional states when learning with self-generated drawings. It was assumed that learners in a positive emotional state would profit from learning with self-generated drawings, while learners in a negative emotional state would not profit from this strategy to the same extent but would rather benefit through reading.

**Methods:**

University students (*N*  =  123) were randomly assigned to one of four conditions resulting from a 2  ×  2 design with self-generated drawings (yes vs. no) and emotional state (positive vs. negative) as independent variables.

**Results:**

Results showed that learning with self-generated drawings was more beneficial for a following transfer test than learning without drawings – irrespective of a learner’s emotional state. The quality of self-generated drawings predicted the learning outcomes of the retention and pictorial test, but not for transfer.

**Discussion:**

Missing effects of emotional states and the missing interaction with self-generated drawings will be discussed.

## 1. Introduction

An overarching goal of learning and instruction is to promote learners in making actively sense of the to-be-learned information. To achieve this goal, several generative learning strategies have been proposed (Fiorella and Mayer, 2022). One of these strategies, which is in the focus of the current study, is the strategy of self-generated drawings. When instructed to self-generate a drawing during learning, learners are provided with a text and asked to draw the main elements and their relations that are described in the text. Overall, recent reviews and meta-analyses concluded that self-generated drawings are an effective and valuable learning strategy (Fiorella and Zhang, 2018; Cromley et al., 2020; Mayer, 2021; Fiorella and Mayer, 2022; Leutner and Schmeck, 2022). Nonetheless, it is also pointed out in these research overviews that boundary conditions need to be examined to derive better guidelines when self-generated drawing are especially beneficial. In the current study, the potential boundary condition of emotional states was investigated. Thereby, it was examined whether the benefit of self-generated drawings compared to reading text may be particularly beneficial when learners are in a positive emotional state, but not beneficial anymore when learners are in a negative emotional state.

Generative learning strategies, such as self-generated drawings, aim at encouraging learners to actively process instructional materials in order to support a deeper understanding of a learning issue. According to the Cognitive Theory of Multimedia Learning (Mayer, 2022) as well as its extension, the Cognitive Affective Theory of Learning with Media (Moreno, 2006; Moreno and Mayer, 2007), three essential processes are involved in active learning: (1) selecting the relevant information, (2) mentally organizing this information, and (3) integrating the newly acquired information with prior knowledge. Especially the processes of organizing and integrating information (i.e., generative processing; Mayer, 2021) are supposed to be stimulated by generative learning strategies and to contribute to a deeper understanding of the content. In a similar way, the Cognitive Load Theory (Sweller et al., 2011) can be used to describe the beneficial effect of generative learning strategies. The Cognitive Load Theory (CLT) – as well as the Cognitive Theory of Multimedia Learning (CTML) and the Cognitive Affective Theory of Learning with Media (CATLM) – state that working memory has a limited capacity that can be overloaded when dealing with new information. Within CLT, three types of cognitive load are usually distinguished (Sweller et al., 2011). Intrinsic cognitive load reflects the load that is imposed by the complexity of a content, which in turn is also a function of a learner’s prior knowledge. Extraneous cognitive load (ECL) results from a suboptimal instructional design and unnecessarily charges the limited capacity of working memory, thereby hindering learning. Germane cognitive load (GCL) is the load that is invested to gain a deeper understanding of the content. GCL, which is analog to generative processing in the terminology of CTML, can only be invested when free working memory resources are available, that is when ICL and ECL do not already exceed the limited capacity of working memory. To conclude, generative learning strategies are in the context of CLT supposed to lead to a higher investment of germane resources, that is GCL.

Next, to these general considerations on how generative learning strategies work, the *Cognitive Model of Drawing Construction* (Van Meter and Garner, 2005; Van Meter and Firetto, 2013) – which is based upon the CTML – refines how self-generated drawings work. Accordingly, after selecting and organizing the textual information in a verbal mental representation, learners need to construct a nonverbal mental representation from the verbal information, and integrate these two representations, in order to being able to transpose the verbal information into the external drawing. Moreover, self-generated drawings are supposed to activate metacognitive processes, such as monitoring and regulation behaviors, when for instance being faced with difficulties during the drawing process. Such experienced difficulties may not only prompt learners to reinspect the text and to carry out generative processes, but also may result in more accurate judgments of the learning process (e.g., Schleinschok et al., 2017).

Self-generated drawing is especially beneficial when learners are able to produce high quality drawings. This is reflected in the prognostic drawing effect (Schwamborn et al., 2010; Leutner and Schmeck, 2022), which proposes that the quality of learners’ drawings predict learners’ performance on later knowledge tests. Note that the quality of learners’ self-generated drawings does not refer to artistic skill or aesthetic appeal, but it refers to the completeness and accuracy of the to-be-learned information in the drawing. One can assume that the completer and more accurate the drawings are, the more the learners were engaged in the abovementioned underlying metacognitive and cognitive processes. It is apparent that constructing self-generating drawings and the associated cognitive and metacognitive processes may take time (Van Meter, 2001; Schmeck et al., 2014; Hellenbrand et al., 2019; Zhang and Fiorella, 2021).

Even though recent reviews and meta-analyses indicate that self-generated drawings have overall a positive impact on learning outcomes, these reviews and meta-analyses also emphasized that this is not always the case (Fiorella and Zhang, 2018; Cromley et al., 2020; Mayer, 2021; Fiorella and Mayer, 2022; Leutner and Schmeck, 2022). Drawing pictures requires cognitive resources (Leutner et al., 2009; Schwamborn et al., 2011) and additional time and effort to apply this learning strategy accurately. A learner’s willingness to invest effort and dedicate resources for self-generating drawings may though also depend on learners’ emotional states – specifically positive compared to negative emotional states – for at least two reasons. First, emotional states may have consequences on the motivation to learn. However, to successfully perform self-generated learning strategies, learners may need a certain amount of motivation to perform these demanding tasks. Second, emotional states may affect how information is processed, which in turn also may affect the comparison of self-generated learning strategies to other learning activities. In the following, we briefly describe emotional states in multimedia learning and its influence on motivation. Thereafter, we will address the just mentioned role of emotional states in learning with self-generated drawings.

An emotion (e.g., surprise) – in contrast to an emotional state – can be characterized as a short-lived intensive feeling with high pleasure/displeasure that is dependent on a specific referent (Bless and Fiedler, 1995). In contrast to an emotion, an emotional state is less intensive and is not necessarily dependent on a specific referent. An emotional state can be characterized as a rather mild feeling that can be described around its valence (positive – negative). In the current study, we investigate the impact of an emotional state – as is often done in multimedia learning research (see Heidig et al., 2015).

The CATLM (Moreno, 2006; Moreno and Mayer, 2007) connects cognitive and affective processes in multimedia learning. Thereby, it is assumed that motivational and affective factors, such as emotional states, can influence the learning processes of selecting, organizing and integrating information (affective mediation assumption). Following, different emotional states may alter generative processing. Similarly, affective factors have also been taken into account recently in the CLT (Plass and Kalyuga, 2019), and are also assumed to alter the investment of GCL.

In the last decade, based upon the CATLM, research about the influence of emotions and emotional states as well as motivation on multimedia learning has become a topic of major interest – with equivocal research findings (e.g., Um et al., 2012; Navratil et al., 2018; Kühl et al., 2019; Münchow and Bannert, 2019; Navratil and Kühl, 2019; Kühl and Münzer, 2021; Plass and Hovey, 2022; for meta-analyses see Brom et al., 2018; Wong and Adesope, 2020). For instance, results of an influential study by Um et al. (2012) showed that learners’ positive emotional state and meaningful learning have a positive relation. In particular, the authors observed that students with an externally induced positive emotional state showed better transfer performance compared to an induced neutral state. Likewise, these students reported higher motivation during learning than students with the induced neutral state (see also Plass et al., 2014). However, there is also contradicting evidence, showing that an externally induced negative emotional state can potentially have positive effects on learning outcomes (Knörzer et al., 2016). These contradicting results, as well as a mixed pattern of results on more basic research about emotional states on learning (Fiedler and Beier, 2014), suggest that it is not easy to draw clear conclusions from the connection between an emotional state and learning success. Rather, it may also depend on the demands of the learning process (for instance of generative processes), whether it is better to learn in a positive or negative emotional state. In the following, we will argue that generative processes may be fostered or hampered, depending on the emotional state.

Learners’ emotional state can influence the learning process (Fiedler, 1991), and it is assumed that emotional state is related to motivation (Liew and Tan, 2016): A positive valence of learners’ emotional state can potentially increase learner motivation. Higher motivation – especially when performing additional generative learning strategies – may help to monitor the quality of and proceed with the generative learning activity. Contrarily, negative emotional states could involve low motivation that may hinder learning, especially with an additional generative learning strategy that requires more resources and effort. Moreover, in a negative emotional state, learners might feel the need to regulate their emotional state and invest effort in emotional regulatory processes rather than in generative and metacognitive processes. Following, generative learning strategies may be particularly beneficial in a positive emotional state due to an increase in motivation to invest germane resources to perform the task.

Next, to this motivational approach, emotional states may also directly influence cognitive processing. Based on theories concerning the influence of emotional state on information processing (e.g., Fiedler, 2001; Fiedler et al., 2003), a positive emotional state should rather support learning with tasks that request cognitive flexibility or the generation of new information. Hence, a positive emotional state may be especially beneficial for tasks that require generative strategies. On the other hand, a negative emotional state triggers a more detailed and rigid manner of processing information (Bless and Fiedler, 1995; Fiedler, 2001; Schukajlow et al., 2021). This may be advantageous for learning presented information, such as a given text, without the requirement of generative strategies.

Some empirical evidence for this line of reasoning comes from a study by Fiedler et al. (2003, Exp. 3) as well as from a study by Schindler et al. (2017). In a nutshell, Fiedler et al. (2003) found that for learners in a positive emotional state, self-generated words were better recalled than experimenter provided words, while the opposite was true for learners in a negative emotional state. Similarly, Schindler et al. (2017) found that concepts, where learners had to generate words of the concepts, were better remembered in a positive compared to a negative emotional state, while this did not hold true for concepts consisting of experimenter provided words.

Applying the abovementioned arguments to self-generated drawings, it can be assumed that learning with self-generated drawings compared to learning with only text may be especially beneficial under a positive emotional state. However, in a negative emotional state, the act of self-generated drawings compared to reading text might backfire, since learners might not be willing to invest the necessary effort to accomplish the self-generating task. Since learners in a negative emotional state tend to process text in a more rigid and detail-oriented fashion (Bless and Fiedler, 1995; Knörzer et al., 2016), it may be speculated that in this case learning with text might even be better suited than learning with self-generated drawings.

The present study investigated the influence of externally induced emotional states (positive vs. negative) on learning with self-generated drawings (yes vs. no). Participants in the self-generated drawing conditions were provided with a predrawn background, since a predrawn background reduces the likelihood to be overwhelmed by the drawing task (Schwamborn et al., 2010; Fiorella and Zhang, 2018). Next to learning outcome measures (retention, transfer and pictorial knowledge), learners’ emotional state, motivation, cognitive load, the quality of learners’ self-generated drawings as well as learning time were assessed.

The primary research question pertained to learning outcomes. An interaction of learners’ emotional state (positive vs. negative) and self-generated drawings (yes vs. no) was assumed (Hypothesis 1): We presumed that learners in a positive emotional state would profit from learning with self-generated drawing compared to learning through reading only (Hypothesis 1a). In contrast, we expected that learners in a negative emotional state would not profit from learning with self-generated drawings compared to learning through reading only (Hypothesis 1b).

The secondary research questions addressed possible mechanism – namely motivation, cognitive load, the quality of the drawings and learning time – that may relate to the assumed effect on learning outcomes. Corresponding to the idea that different emotional states are accompanied with varied motivation levels we assumed that learners in a positive emotional state would report higher motivation to learn the instructional material compared to learners in a negative emotional state (Hypothesis 2). In addition, we examined whether the assumed effects on learning outcomes would be mirrored by respective changes in cognitive load, namely invested effort, concentration, but also in perceived difficulty as well as in metacognitive accuracy (here feeling of success). In case self-generated drawing would mainly initiate generative processing (i.e., GCL) for learners in a positive emotional state, then more concentration and invested effort (as a proxy for GCL) for self-generated compared to no drawings may only be observable for learners in a positive emotional state, but not for learners in a negative emotional state (Hypothesis 3). Further it was explored whether self-generated drawings would lead to an increase in perceived difficulty, as the drawing task is demanding, and connected to a more accurate estimations of feeling of success – and whether these potential effects would be altered by learners’ emotional state.

Furthermore, it was examined whether a positive compared to a negative emotional state would lead to a higher quality of the drawings that were produced during learning (Hypothesis 4a). Connected, according to the prognostic drawing effect, we assumed that the quality of the drawings should predict learning outcomes. That is, the higher the quality of the drawings is, the better the outcomes in the knowledge tests will be (Hypothesis 4b). Finally, we examined the influence of time on task. Based on previous research, we assumed that learning with self-generated drawings would take more time than learning through reading only. Further, we explored whether the expected pattern of results for learning outcomes would also be mirrored by the time learners spent with the instructional material – and if so, whether time on task would be suited to explain the observed findings.

## 2. Materials and methods

### 2.1. Participants and design

A total of 133 students from a German University volunteered to participate for either course credit or payment. The data of 10 students had to be excluded from further analyses: Two learners in the drawing condition did not follow the drawing instructions, seven learners generated drawings in the no-drawings condition and one student spoke German for only 3 years and indicated problems in understanding the German instructional material. The remaining 123 learners (88 female/35 male, average age, 21.34 years, *SD* = 2.91) were randomly assigned to one of four conditions, resulting from a 2 × 2 between-subject design with learners’ emotional state (positive vs. negative) and self-generated drawing (yes vs. no) as independent variables (see Table 1).

**Table 1:** Means (and SD) for emotional state, learning outcome, motivation, quality of the drawings, cognitive load and learning time in the four experimental conditions.

Emotional state	Positive		Negative	
Self-generated drawings	No-drawing (*n* = 29)	Drawing (*n* = 32)	No-drawing (*n* = 30)	Drawing (*n* = 32)
**Emotional state manipulation checks**				
After film segment	5.45 (0.95)	5.47 (1.27)	2.67 (0.92)	2.59 (1.10)
After learning phase	3.97 (0.82)	4.19 (1.38)	4.23 (0.82)	4.22 (1.18)
**Learning outcomes**				
Retention	5.76 (1.79)	5.75 (2.31)	5.42 (2.45)	6.28 (2.60)
Transfer	1.38 (1.12)	1.88 (1.34)	1.37 (0.96)	1.91 (1.00)
Pictorial test	3.50 (1.49)	3.95 (1.40)	3.17 (1.45)	3.50 (1.26)
**Motivation**				
Motivation	30.31 (9.99)	32.63 (11.71)	33.50 (10.91)	31.78 (9.47)
**Quality of self-generated drawings**				
Drawing score	---	12.63 (2.01)	---	13.08 (2.11)
**Cognitive load**				
Effort	5.07 (1.41)	4.84 (1.35)	4.77 (1.19)	4.97 (1.36)
Difficulty	3.17 (1.34)	3.00 (1.30)	2.33 (1.27)	3.41 (1.16)
Concentration	5.24 (0.99)	5.28 (1.17)	5.03 (1.03)	5.37 (1.19)
Feeling of success	3.90 (1.18)	4.25 (1.02)	4.23 (0.94)	4.22 (1.07)
**Learning time**				
Time in seconds	509.24 (167.04)	739.06 (234.54)	533.73 (174.76)	839.97 (338.27)

### 2.2. Emotional state induction

Emotional states can successfully be induced by watching valence-loaded film segments (Westermann et al., 1996). Two video clips were chosen that were successfully applied by a study from Navratil and Kühl (2019): ‘Ice Age – Trailer 3’ (approx. 02:09 min) to induce a positive emotional state and ‘Bambi (the part where Bambi’s mother died)’ (approx. 02:13 min) to induce a negative emotional state. These two film segments were used for the current study.

### 2.3. Instructional material

The text-based instructional material was adapted from Mayer and Moreno (1998) as well as Mayer (2021) and dealt with the topic of lightning formation. There were two different versions: (1) a text-only condition (no-drawing condition) and (2) a condition with identical text and always the same provided predrawn backgrounds for generating pictures (self-generated drawings condition). The text consisted of 298 words that were separated in six paragraphs and distributed over three printed pages (DIN A4, landscape format). In the drawing condition, the text was provided on the left side and all provided predrawn backgrounds were presented on the right side. The predrawn background was based on the illustrations provided by Mayer (2021), but solely contained at the bottom a ground with a house and two trees and at the top a symbolized freezing level in the sky. Learners were instructed to draw the most important concepts of the respective text paragraph into these provided predrawn backgrounds. Learners in the no-drawing condition received the same instructional material, with the crucial exception, that the right side remained blank.

### 2.4. Measures

The measures consisted of (1) prior knowledge questions, (2) emotional state manipulation checks, (3) cognitive load items, (4) a motivation questionnaire, (5) a knowledge test to measure learning outcomes, (6) the coding concerning the quality of the to be generated drawings, and (7) the time spent with the instructional material. For explorative reasons, (8) a need for cognition questionnaire (Bless et al., 1994), (9) a learning style questionnaire (Santa Barbara Learning Style Questionnaire & Verbal-Visual Learning Style Rating; Mayer and Massa, 2003), and (10) evaluative questions were administered after the knowledge test. A (11) spatial ability test (Paper Folding Test; Ekstrom et al., 1976) and a demographical questionnaire were assessed in an unrelated study a week earlier. Measures that served for explorative reasons were not in the scope of this article and will not be further explored (also due to space limitations), but will at least be reported as control variables in the results section.

#### 2.4.1. Prior knowledge questions

Learners were asked to assess their previous knowledge of the topic ‘weather’ on a 5-point scale (from ‘very little’ to ‘very much’). Furthermore, adapted from Mayer and Moreno (1998), learners had to assess their knowledge about six relevant weather-related items (e.g., ‘I know what a cold front is’). A summed score, defined as ‘experience score’, was generated by tallying the number of relevant weather-related items and adding the number to the level of knowledge the learners estimated on the 5-point scale (min = 1, max = 11). Moreover, learners were asked to write down everything they know about lightning formation, which is termed ‘prior knowledge’ in the following. This prior knowledge question was identical to the retention question of the knowledge test – with the exception that answering the prior knowledge question was without time restriction – and was scored the same way (see below in section 2.4.5).

#### 2.4.2. Emotional state manipulation checks

To examine learners’ emotional state, the emotional state manipulation check had to be rated on a 7-point scale: ‘Right now, I am feeling…’ (from ‘extremely depressed’ to ‘extremely happy’). This manipulation check was assessed twice: the first time directly after the emotional state induction and the second time directly after the learning phase. The first time, learners were asked in total five questions, to make the check look inconspicuous. At this the manipulation check was the third question. The remaining four questions, that also had to be rated on a 7-point scale, served as filler items and asked about the interestingness of the video clips, how well participants could concentrate on the clips, whether the clip improved or impaired participants mood, and whether they would have liked to see more of the video clip. For the second emotional state manipulation check, only the first item described above was utilized and was intertwined with the cognitive load and feeling of success items.

#### 2.4.3. Cognitive load and feeling of success

Three items were used to assess learners’ cognitive load when learning with the instructional material: One item referred to invested mental effort (“How much mental effort did you invest?”; *cf.* Paas, 1992), one item referred to difficulty (“How difficult was it to learn with the material?”), and one item referred to concentration (“How much did you concentrate during learning?”; *cf.* Cierniak et al., 2009). Moreover, learners’ feeling of success in the subsequent knowledge test was assessed (“How successful do you think you will be in answering a knowledge test?”). All items had to be rated on a 7-point scale (from 1 = ‘not at all’ to 7 = ‘very much’).

#### 2.4.4. Motivation

A questionnaire from Isen and Reeve (2005), which was translated into German, was used. It consisted of eight items that asked about the instructional material (e.g., “It stimulates my curiosity.”) that had to be rated on a 7-point scale (from 1 = ‘strongly disagree’ to 7 = ‘strongly agree’; α = 0.92).

#### 2.4.5. Knowledge test

Learning outcomes were measured by means of (1) a *retention*, (2) a *transfer* and (3) a *pictorial* test. For the retention test, learners had to write down everything they could remember. According to a scoring scheme, learners received one point for each of 16 major idea units that they included in their answer to the retention question, regardless of wording (observed maximum score: 15 points). The transfer test comprised three questions which always referred to the most important steps of lightning (Mayer and Moreno, 1998). The three questions were: (1) “What could be done to decrease the intensity of a lightning storm?,” (2) “Suppose you see clouds in the sky, but no lightning. Why not?,” (3) “What does air temperature have to do with lightning?” To solve the transfer test, learners had to apply their acquired knowledge to new scenarios. Each transfer question was scored according to a coding scheme, which consisted of a list of possible correct answers. Acceptable answers to the first question would for instance be “placing negative particles to the earth surface” or “placing positive particles near the cloud.” Acceptable answers to the second question would for instance be “the cloud may be below the freezing level” or “no positive and negative charges have built up in the cloud yet.” Acceptable answers to the third question would for instance be “the top of the cloud is above freezing level and the bottom of the cloud below freezing level” or “the air temperature is cooler than the ground.” The final score for transfer was determined by summing up the points from all transfer questions (observed maximum score: four points). The pictorial test consisted of five provided predrawn background pictures. Learners were instructed to draw the most relevant aspects of lightning in these background pictures. These test items (Schmidt-Weigand and Scheiter, 2011) were similar to the provided partly predrawn backgrounds in the drawing condition. For each appropriate and identifiably drawn object, a half point was awarded and summed up to one score (observed maximum score: six points). The inter-rater reliability was good for retention (*r* = 0.88, *p* < 0.001), moderate for transfer (*r* = 0.73, *p* < 0.001) and good for the pictorial test (*r* = 0.85, *p* < 0.001). Cases of disagreement were resolved by reaching a consensus.

#### 2.4.6. Quality of self-generated drawings

Learners in the condition self-generated drawing were instructed to draw the most important concepts of the six text paragraphs into the six provided predrawn backgrounds. The quality of the self-generated drawings refers to the completeness and accuracy of the to-be-learned information in the drawing (i.e., correctness), but not to the aesthetic appeal. A coding scheme for scoring learners’ correctness of the self-generated drawings during learning was developed. At this, for each of the six drawings, two to four aspects were relevant and coded (max = 17 points). The drawings were corrected by two independent raters. Interrater-agreement was moderate (*r* = 0.73, *p* < 0.001) and cases of disagreement were resolved by reaching a consensus.

#### 2.4.7. Learning time

The time participants had spent on each computerized page in the web-based learning environment (UNIPARK) was logged. During working with the paper-based material, the computerized page explaining the instruction for the learning phase was presented. Following this site, there was an additional computerized page that asked participants, whether they finished the learning phase and if so, that they should click the continue-button. This additional page was implemented in case participants should accidently click on the continue-button on the previous page (which was occasionally the case). It was possible to retrace the time participants spent with the paper-based instructional material by summing up the total time participants stayed on these two pages (in seconds). Note however, that this procedure is rather an estimation of the time learners actually spend with the instructional material.

### 2.5. Procedure

**Figure 1:**
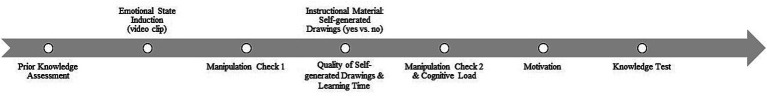
An overview of the basic procedure. The gray arrow represents the course of time. The boxes above the gray arrow represent the two independent variables, the boxes below the arrow represent the assessed measures.

A graphical outline about the basic procedure is given in Figure 1. In each session, up to six learners took part. In one session, only learners who were asked to draw or only learners who were not asked to draw took part – to avoid unnecessary conspicuousness (e.g., drawing noises for learners in the no-drawing condition). Each learner had an individual computer and headphones. Learners received computerized as well as paper-based material. The computerized material was presented (and the data logged) by the web-based survey software UNIPARK.^1^ Participants were informed, via a written instruction on the screen, that there are two sub-studies: (1) Rating a film segment and (2) learning with specific learning material. The actual purpose of the study was not explained to avoid demand effects. Then, learners were asked to fill out the computerized prior knowledge questionnaire (without time restrictions). Thereafter, all learners listened to a piece of relaxing music to ensure a neutral emotional baseline, followed by the emotional state induction (presenting randomly either a positive or a negative film segment). Subsequently, participants were asked to fill out the first manipulation check. Then, the learning phase started. At this, learners read the instruction that they should give a sign to the instructor, so that the instructor would then hand out the paper-based instructional material to them. They also read the instruction that there were no time restrictions to learn with the instructional material and that when they think that they have finished learning, they should again give a sign so that the instructor could collect the paper-based instructional material. When collecting the paper-based instructional material, the instructor asked participants to click the continue-button in the learning environment. Note that there was an additional computerized page that asked participants, whether they finished the learning phase and if so, that they should click the continue-button. Then, participants had to fill out the second manipulation check, the cognitive load items, and the motivation questionnaire on the computer, followed by working on the paper-based knowledge test. Each question of the knowledge test was time-restricted: Four minutes for the retention test, 3 min for each of the three transfer tasks, and 8 min for the pictorial test. Participants were instructed to wear headphones during working on the paper-based knowledge and it was announced via headphones when time was up for each question. Finally, learners were instructed to fill out the NFC questionnaire, the learning style questionnaire and the evaluative questions (one participant did not fill out the latter questionnaires). A single session lasted about 45 min. Participants in this study had already participated in an unrelated study a week earlier, in which spatial abilities and demographic data were collected. By assigning a study participant number, the data could then be merged.

An ethics approval by means of an ethical board was not mandatory, neither by the University’s guidelines nor by national regulations in Germany and was not requested. Nevertheless, there are ethical guidelines of the German Psychological Society’s (DGPs; 2004, CIII) and the whole conducted experiment followed the rules set by these ethical guidelines. All subjects were aware of taking part in research. Before starting the experiment, each participant received a written informed consent, where they were informed about the possibility of quitting the experiment with no repercussions or disadvantage at any time. All participants signed the informed consent and allowed us to use their collected data anonymously for research purposes.

### 2.6. Data analyses

To be able to examine Hypotheses 1–3, we conducted two-factorial ANOVAs with self-generated drawings (yes vs. no) and emotional state (positive vs. negative) as independent variables for the dependent variables emotional state manipulation check, learning outcomes, motivation, cognitive load and feeling of success (as well as for the control variables). Only learners in the drawing condition could be considered for the analyses of the dependent variable quality of self-generated drawings. Therefore, we conducted a *t*-test for independent means between the two conditions positive emotional state and negative emotional state to be able to answer Hypothesis 4a. Furthermore, we related the quality of self-generated drawings to learning outcomes by correlational analyses to be able to answer Hypothesis 4b. To examine the influence of time on task, we conducted two-factorial ANOVAs with self-generated drawings (yes vs. no) and emotional state (positive vs. negative) as independent variables for the dependent variable learning time. To explore whether time on task would be suited to explain the observed findings, we conducted a mediation analysis with the mediator learning time.

## 3. Results

All descriptive data of the dependent variables are presented in Table 1. As a measure of effect size, partial eta-squared (ŋ*^2^_p_*) is reported. For eta-squared, effect sizes of 0.01, 0.06, and 0.14 correspond to small, medium, and large effect sizes, respectively (*cf.* Cohen, 1988).

### 3.1. Control variables

For the control variables experience score, prior knowledge, as well as need for cognition, learning style and spatial abilities, two-factorial ANOVAs with self-generated drawings (yes vs. no) and emotional state (positive vs. negative) revealed no main effects for emotional state or for self-generated drawings, and no interaction of emotional state and self-generated drawings (all *F_s_* < 2.64, all *p_s_* > 0.10), indicating that the randomization was successful.

### 3.2. Emotional state manipulation checks

After viewing a film segment, there was a significant effect for emotional state, *F*(1, 119) = 212.46, *p* < 0.001, ŋ*^2^_p_* = 0.641: Learners viewing the positive film segment reported a higher positive emotional state (*M* = 5.46, *SD* = 1.12) compared to learners viewing the negative film segment (*M* = 2.63, *SD* = 1.01). The value of four would represent the middle of the scale. The results showed neither a main effect of self-generated drawings nor an interaction of emotional state and self-generated drawings (both *F_s_* < 1, both *p_s_* > 0.10, both ŋ*^2^_p_* < 0.001). These findings show that the intended emotional state induction worked out. However, as indicated by the second manipulation check, the differences in emotional state between conditions was no longer present after the learning phase ended. Results did not reveal neither a main effect of emotional state anymore and also (still) no effect of self-generated drawings, or an interaction of both factors (all *F_s_* < 1, all *p*_s_ > 0.10, all ŋ*^2^_p_* < 0.005).

### 3.3. Learning outcomes

The main hypothesis of this study states that learners` emotional states influence learning with self-generated drawings in the way that, learners learning with a positive emotional state profit from the learning with self-generated drawings (Hypothesis 1a), whereas learners in a negative emotional state profit from this strategy not in that extent but may even rather profit from learning by reading (Hypothesis 1b). Two-factorial ANOVAs were performed for retention, transfer and the pictorial test. For the retention test, results yielded neither a significant main effect of self-generated drawings, nor of emotional state, nor an interaction (all *F_s_* < 1.1, all *p*_s_ > 0.10, all ŋ*^2^_p_* < 0.010). For the transfer test, results yielded a significant main effect of self-generated drawings, *F*(1, 119) = 6.61, *p* = 0.01, ŋ*^2^_p_* = 0.053: Learning with self-generated drawings (*M* = 1.89, *SD =* 1.17) resulted in better learning outcomes than learning without it (*M* = 1.37, *SD* = 1.03). Further results yielded neither a significant main effect of emotional state, nor an interaction (both *F_s_* < 1, both *p*_s_ > 0.10, both ŋ*^2^_p_* < 0.001). For the pictorial test, results yielded neither a significant main effect of self-generated drawings, *F*(1, 119) = 2.42, *p* = 0.12, ŋ*^2^_p_* = 0.020, nor of emotional state, *F*(1, 119) = 2.42, *p* = 0.12, ŋ*^2^_p_* = 0.020, nor an interaction,, *F* < 1, *p* = 0.81, ŋ*^2^_p_* < 0.001. For transfer, Hypothesis 1b has to be rejected since learners in a negative emotional state also profited from self-generated drawings, just like learners in a positive emotional state (in accordance with Hypothesis 1a).

### 3.4. Motivation

Regarding motivation, a 2 × 2 ANOVA showed neither a significant main effect of self-generated drawings, nor of emotional state, nor an interaction between the two factors (all *F_s_* < 1.2, all *p*_s_ > 0.10, all ŋ*^2^_p_* < 0.010). Thus, Hypotheses 2 has to be rejected.

### 3.5. Quality of self-generated drawings

For the analyses of the self-generated drawings, only the 64 learners in the drawing condition could be considered. It was assumed that different emotional states might influence the quality of the drawings (Hypothesis 4a). However, the quality of the self-generated drawings was not influenced by learners’ emotional states, *t*(62) = 0.88, *p* = 0.38, *d* = 0.22. This result corresponds to the reported finding that there was no influence of emotional states on motivation or learning outcome. Partly in line with the prognostic drawing effect (Hypothesis 4b), a significant correlation was found between the quality of the self-generated drawings and retention scores, *r* = 0.27, *p* = 0.03, and the pictorial test, *r* = 0.32, *p* = 0.009, but not for the transfer test, *r* = 0.006, *p* = 0.96.

### 3.6. Cognitive load and feeling of success

Also opposed to Hypothesis 3, for invested effort, concentration as well as feeling of success, a 2 × 2 ANOVA revealed neither a significant main effect of self-generated drawings, nor of emotional state induction, nor an interaction of these factors (all *F_s_* < 1, all *p*_s_ > 0.10, all ŋ*^2^_p_* < 0.010). Regarding perceived difficulty, results revealed no significant main effect of emotional state, *F* < 1, *p* > 0.10, ŋ*^2^_p_* < 0.010. However, a marginal main effect of self-generated drawings was observed [*F*(1, 119) = 3.89, *p* = 0.051, ŋ*^2^p* = 0.032]: Learning with self-generated drawing was perceived as more difficult (*M* = 3.20, *SD* = 1.24) than as learning without them (*M* = 2.75, *SD* = 1.36). Also, a significant interaction of self-generated drawings and emotional state was found for difficulty [*F*(1, 119) = 7.43; *p* = 0.007, ŋ*^2^p* = 0.059]: Learners receiving a negative emotional state induction experienced the instructional material with self-generated drawings as more difficult (*M* = 3.41, *SD* = 1.16) than reading (*M* = 2.33, *SD* = 1.27), *p* = 0.001. For learners receiving a positive emotional state induction, however, both self-generated drawing conditions were experienced as equally difficult, *p* = 0.60.

### 3.7. Learning time

Regarding the time spent with the instructional material, a 2 × 2 ANOVA showed a main effect for the factor self-generated drawings, *F*(1, 119) = 37.92, *p* < 0.001, ŋ*^2^_p_* = 0.242, with learners who generated drawings spending more time with the instructional material (*M* = 789.52, *SD =* 293.19) than learners who learned without self-generated drawings (*M* = 521.69, *SD* = 169.98). There was no main effect of emotional state observable, *F*(1, 119) = 2.08, *p* = 0.15, ŋ*^2^_p_* = 0.017, as well as no interaction between emotional state and self-generated drawings, *F* < 1, *p* > 0.10, ŋ*^2^_p_* < 0.010.

The longer learning times of learners in the condition self-generated drawings compared to learners in the condition without self-generated drawings mirrored their better performance in the transfer test. To examine whether time spent with the instructional material could already explain the better performance in the transfer test when learning with self-generated drawings, we conducted a mediation. Thereby, self-generated drawings (yes vs. no) served as independent variable, time spent with the instructional material as mediator and transfer performance as dependent variable. The SPSS-macro Process v4.3 (Hayes, 2022) was used with 5,000 bootstrap samples. There was no effect of the mediator variable learning time on transfer performance *b* = 0.0001, 95% CI [− 0.0008, 0.0009], and the indirect effect was not significant, *b* = 0.137, 95% CI [− 0.2161, 0.2282], meaning that the differences between conditions concerning the time spent with the instructional material could not explain the differences in transfer performance.

## 4. Discussion

The present study investigated whether learners’ emotional state would moderate learning with self-generated drawings. We assumed that only a positive emotional state, but not a negative emotional state, would foster learning with self-generated drawings compared to no drawings – and that this would be reflected in learning outcomes, and associated also in motivation, cognitive load and the quality of self-generated drawings. However, other than assumed for learning outcomes (Hypothesis 1), for both, learners in a positive as well as learners in a negative emotional state, self-generated drawings were beneficial to achieve a deeper understanding of the content (i.e., transfer test). Even though participants spent more time when self-generating drawings than when not generating drawings, time on task was not suited to explain the beneficial effect of self-generated drawings. Also concerning motivation, cognitive load and the quality of self-generated drawings, no effects of learners’ emotional state, but also no effects of self-generated drawings were observed (opposed to the accompanying Hypotheses 2, 3, and 4a) – with the exception of perceived difficulty, which will be discussed below. However, results confirmed the prognostic drawing effect partially (Hypothesis 4b): The quality of the drawings was predictive for learning outcomes of retention and the pictorial test but not for transfer. Overall, the observed results speak against the assumption that generative learning strategies are influenced by a learner’s emotional state, questioning the transferability of rather basic research (Fiedler et al., 2003) to more applied multimedia learning scenarios (see also Navratil et al., 2018; Navratil and Kühl, 2019). In the following, the results and missing effects will be discussed in more detail with respect to the dependent variables.

### 4.1. Emotional state

The emotional state induction procedure was successful: Learners who viewed a positive film segment reported a more positive emotional state than learners who viewed a negative film segment, with a very large effect size. However, the emotional state appeared not to persist over the learning phase: Learners did not significantly differ in their subjective rating of emotional state irrespective of initial differences in emotional state. According to Gross and Levenson (1995), it may be the case that this kind of induction procedure lasts only for several minutes. Moreover, it could be that the effect of the induction vanishes because of learners’ shift of attention to the instructional material (Fiedler et al., 2003).

### 4.2. Learning outcomes

Concerning learners’ retention knowledge, it seems that neither their emotional state nor self-generated drawings nor both factors together have an influence. However, it is argued that the effectiveness of generative learning strategies, such as self-generated drawings, may not necessarily be expected for retention, but particularly for transfer tasks asking for a deeper understanding of the content (Fiorella and Zhang, 2018).

With respect to the transfer knowledge, learners with self-generated drawings outperformed learners learning by reading. This result speaks in favor for the effectiveness of self-generated drawings. Although this result is in line with several studies, it cannot be taken for granted (Fiorella and Zhang, 2018; Cromley et al., 2020; Leutner and Schmeck, 2022). In this study, the instructional material was used the first time in the context of self-generated drawings. Thereby, this study contributes to the generalizability of the effect of self-generated drawings on learning and extends previous work.

Regarding the pictorial test, it seems that neither learners’ emotional state nor self-generated drawings nor both factors together had an influence. No main effect of self-generated drawings is quite unexpected because learners had the opportunity to exercise this task already in the self-generated drawings condition, whereby the provided partly predrawn backgrounds in the learning phase and in the pictorial test were very similar. One possible explanation may be that the assessed pictorial test may lack sensitivity: Schmidt-Weigand and Scheiter (2011) only found a superiority of text and pictures over text for this test when the text did not contain spatial descriptions. However, when the text contained spatial descriptions, there were no differences between learners receiving text and pictures compared to learners receiving only text. Since in our study the text of the used instructional material contained spatial descriptions, this may explain the lack of an effect.

Finally, the missing effect of emotional state for all three types of knowledge test questions might be explained by the decline of induced emotional states during learning, thereby weakening the potential impact of different emotional states on learning. However, it should also be noted that the impact of emotional states on learning outcomes is not always that stable and straightforward (e.g., Plass et al., 2014; Heidig et al., 2015; Knörzer et al., 2016; Navratil et al., 2018; Navratil and Kühl, 2019). In addition, the rather strong experimental manipulation of drawing might have overshadowed the rather subtle experimental manipulation of inducing emotional states. This may generally explain why the observed moderating role of emotional states on generative learning activities, which are found in more basic research (e.g., Schindler et al., 2017), are not easily transferable to more applied educational settings with rather strong experimental manipulations (see also Navratil et al., 2018; Navratil and Kühl, 2019) – for learning outcome measures and related also for assumed possible mechanisms.

### 4.3. Motivation

We assumed that learners in a positive emotional state would report higher motivation to learn than learners in a negative emotional state. This relation was shown in a study by Um et al. (2012) in which learners reported higher motivation with an internal or an external positive emotional induction rather than with a neutral one. However, this relation could not be shown in the current study. Even though we used the same questionnaire as Um et al. (2012), it may be the case that the motivation questionnaire was not optimal to detect the potential impact of emotional states on motivation for the current study. More precisely, learners firstly viewed a positive or negative film segment, and thereafter had to learn with this instructional material. However, the questionnaire asked how motivated they were to learn with this instructional material. Hence, it might be the case that they mainly judged the instructional material and not their overall motivation to learn, which in turn may have been influenced by their initial emotional state. Moreover, the motivational questionnaire was assessed after the learning phase, when no differences in emotional states between learners in the different conditions were observable anymore. This also might have overshadowed a potential influence of emotion on motivation – in case this relationship existed in this study.

### 4.4. Quality of self-generated drawings

As for learning outcomes, motivation and cognitive load, a learner’s emotional state also had no impact on the quality of self-generated drawings (as opposed to Hypothesis 4a). Regarding retention as well as the pictorial test, in line with several studies (see Leutner and Schmeck, 2022), a positive relation with the quality of the drawings was demonstrated, confirming the ‘prognostic drawing effect’ for these dependent measures. However, no relationship between the quality of the self-generated drawings and the learning outcomes of the transfer test could be shown. This result is at first glance unexpected, but may be well explained: When learning with self-generated drawings, generative processes are performed (Leutner and Schmeck, 2022). These processes take place during learning and enhance deeper understanding. However, not all of those generative processes are directly reflected in the quality of the resulting drawings (particularly if drawings are assessed quite directly by the elements included). Rather, the drawing task asks mainly for drawing concepts of factual knowledge, but does not necessarily reflect a deeper engagement with the instructional material. Accordingly, only for the learning outcomes measures that were asking for facts, namely retention and pictorial knowledge, a correlation between drawings and test scores were found, whereas the effect of self-generated drawings on deeper understanding was not apparent in the drawings.

### 4.5. Cognitive load

Learners perceived the instructional material as more difficult when they were asked to generate drawings compared to when they were not asked to generate drawings. This was however only the case when a negative emotional state was induced but not when a positive emotional state was induced. At first glance, this may be interpreted as partly mirroring the supposed interaction, namely that self-generated strategies can be detrimental to learning when learners are in a negative emotional state. However, the learning outcome measures do not support this conclusion; rather, self-generated drawings were also beneficial for learners in a negative emotional state. Given that learners perceiving self-generated drawings as more difficult, and that learning with self-generated drawings led to a better transfer performance, they may be subsumed to the framework of desirable difficulties (Bjork and Bjork, 2011). Concerning the other subjective ratings of invested effort, concentration as well as feeling of success, no differences between conditions were observable. It may be the case that assessing these variables by single item self-report data is not the best approach, and that more sensitive and reliable instruments are needed (Klepsch and Seufert, 2020).

### 4.6. Learning time

Learners clearly spent more time when they were instructed to self-generate drawings than when they were instructed to learn by reading. This may be well explained by the fact that the act of generating a drawing as well as the assumed corresponding cognitive and metacognitive processes need time (Van Meter, 2001). The research on whether the beneficial effect of self-generated drawings can be traced back solely to time on task is equivocal (*cf.* Van Meter, 2001; Schmeck et al., 2014; Hellenbrand et al., 2019; Zhang and Fiorella, 2021) and we did not observe a mediating effect in the current study; further, time on task was not substantially associated with the quality of self-generated drawings. It may be agreed upon that time on task may rather be as a necessary prerequisite to draw and perform cognitive and metacognitive processes, but not sufficient to explain the beneficial effect of learning with self-generated drawings.

We think that discussing time on task is especially important when methods that need more time do not result in a better understanding of the content (same effectiveness, but lower efficiency). However, we also think that this discussion may sometimes be misleading, particularly when methods that bring learners to actually elaborate on contents, and thereby spending more time with them, result in a better understanding (being more effective, even though they may not be more efficient).

### 4.7. Limitations and implications for further research

In the current study, we used a sample size (*N* = 123) that should have been suitable to find the hypothesized interaction of emotional state and self-generated learning for a medium effect size (given a Type I error of 0.05 and a Type II error of 0.20). The sample size was indeed sufficient to find a positive effect of self-generated drawings on transfer performance, with a medium effect size that is somewhat in line with the ones observed in a recent meta-analysis on learning with self-generated drawings (Cromley et al., 2020). However, concerning the hypothesized interactions of emotional state and self-generated learning, the observed effect sizes for learning outcomes and almost all of the other dependent variables were (very) small. Based on these observed effect sizes, it is fair to state that the assumed moderating role of emotional states can be rather neglected for more applied and educational meaningful scenarios – at least as implemented in the current study.

In psychological research, time on task is often fixed so that it cannot have a confounding influence. Thereby, the internal validity is often increased at costs of ecological validity. For the current study we refrained from fixing time on task, since it may produce artifacts: Either, we could have set the time in a manner that it would have been adequate to learn with self-generating drawings; in this case, however, we would have forced learners who did not learn with self-generated drawings to spent more time than they are usually willing to invest. This however is problematic, since it for instance provoke feelings of boredom or frustration or it may result in a longer delay between the learning phase and the test phase, that in turn could interfere with the learning process. Or we could have set the time in a manner that it would have been adequate to learn without self-generating drawings; in this case the potential of self-generated drawings would not unfold due to a lack of time, making the investigation somewhat absurd. Summing up, we think that for the current study it was more appropriate to let learners decide how long they want to work with the instructional material than having time on task fixed.

A limitation of the current study is the duration of externally induced emotional states. Even though the induction of emotional state worked successfully, it did not persist. One solution for this problem could be to refresh the emotional state induction during learning or to use state induction procedures that are present during learning, such as differently valence-loaded music. However, these procedures may have their own drawbacks by interfering with the learning process. Another promising way of an internal induction procedure might lie in using emotional design (Um et al., 2012; Plass et al., 2014; Brom et al., 2018; Wong and Adesope, 2020). At this, various design features, such as baby-face biases and different facets of colors, are integrated in the instructional material to induce a desired emotional state that sustains over the whole learning phase. Even though providing emotional enriched pictures is not very suited to investigate self-generated drawings (where usually no pictures are provided), it may generally be suited to investigate the influence of emotional states on generative learning strategies (Navratil et al., 2018).

In the current study, the scope was on the influence of the valence of emotional states on generative learning strategies. Besides valence, also activation can be triggered by viewing valence-loaded film segments. Accounting for this factor, activation could be additionally assessed in future studies. Even though self-generated drawings were beneficial in the current study, their potential might not have been exploited. Since self-generated drawings can be incomplete or erroneous, it may be beneficial if learners would receive constructive feedback of their performance (Chi, 2009). Furthermore, because the self-generated drawing strategy can be very unfamiliar, a pre-training could be offered in which learners learn how to use this learning strategy most effectively and have the opportunity to practice it.

## 5. Conclusion

To conclude, the initial idea of this study was – also based on more basic research (Fiedler et al., 2003; Schindler et al., 2017) – to examine whether self-generated learning activities, in this instance self-generated drawings, would be particularly beneficial when learners were in a positive emotional state, but not beneficial anymore when learners were in a negative emotional state. Whereas the main research question pertained to learning outcomes, additionally possible mechanism (motivation, cognitive load, the quality of the drawings and learning time) were surveyed. However, other than expected, learners’ emotional state had no impact on learning with self-generated drawings compared to no drawings for any learning outcome measure. In line with this finding, and somewhat consequently, learners emotional state also had essentially no impact on the assumed possible mechanisms of motivation, cognitive load, learning time and quality of self-generated drawings. This finding, along with other findings (Navratil et al., 2018; Navratil and Kühl, 2019), questions whether the observed effect in more basic research is transferable to more applied and educational meaningful settings. Besides the missing moderating role of emotional state, there was basically also no main effect of learners’ emotional state on learning outcome measures, motivation, cognitive load, learning time and the quality of self-generated drawings. This finding is overall not in line with findings from recent meta-analyses (*cf.* Brom et al., 2018; Wong and Adesope, 2020). However, there are several studies that also hardly find a (straightforward) impact of emotional states on learning (e.g., Plass et al., 2014; Heidig et al., 2015; Knörzer et al., 2016; Navratil et al., 2018; Navratil and Kühl, 2019), indicating that there is no simple relationship of emotional states on learning. Irrespective of that, this study showed a beneficial effect of self-generated drawings on transfer knowledge, which is in line with a recent meta-analysis as well as review on this topic (Fiorella and Zhang, 2018; Cromley et al., 2020). Additionally, results confirmed the prognostic drawing effect for retention and pictorial knowledge (*cf.* Leutner and Schmeck, 2022). Overall, the study contributed to the research about the impact of self-generated drawings as a promising learning strategy that should be further researched.

## Data availability statement

The raw data supporting the conclusions of this article will be made available by the authors, without undue reservation.

## Author contributions

SN: Writing-Original draft, Writing-Reviewing and Editing, Conceptualization, Methodology, Investigation, Validation, Formal Analysis, Resources, Project administration TK: Writing-Reviewing and Editing, Conceptualization, Methodology, Validation, Formal Analysis, Resources, Data Curation, Funding Acquisition, Supervision, Project administration.

## Footnotes

^1^ www.unipark.com
